# Interaction of Salivary alpha-Amylase and Amylase-Binding-Protein A (AbpA) of *Streptococcus gordonii* with Glucosyltransferase of  *S. gordonii* and *Streptococcus mutans*

**DOI:** 10.1186/1471-2180-7-60

**Published:** 2007-06-25

**Authors:** Biswendu Chaudhuri, Jennifer Rojek, M Margaret Vickerman, Jason M Tanzer, Frank A Scannapieco

**Affiliations:** 1 Department of Oral Biology, School of Dental Medicine, State University of New York at Buffalo, New York, USA; 2 Department of Periodontics and Endodontics, School of Dental Medicine, State University of New York at Buffalo, New York, USA; 3 Department of Oral Health and Diagnostic Sciences, School of Dental Medicine, University of Connecticut Health Center, Connecticut, USA

## Abstract

**Background:**

Glucosyltransferases (Gtfs), enzymes that produce extracellular glucans from dietary sucrose, contribute to dental plaque formation by *Streptococcus gordonii *and *Streptococcus mutans*. The alpha-amylase-binding protein A (AbpA) of *S. gordonii*, an early colonizing bacterium in dental plaque, interacts with salivary amylase and may influence dental plaque formation by this organism. We examined the interaction of amylase and recombinant AbpA (rAbpA), together with Gtfs of *S. gordonii *and *S. mutans*.

**Results:**

The addition of salivary alpha-amylase to culture supernatants of *S. gordonii *precipitated a protein complex containing amylase, AbpA, amylase-binding protein B (AbpB), and the glucosyltransferase produced by *S. gordonii *(Gtf-G). rAbpA was expressed from an inducible plasmid, purified from *Escherichia coli *and characterized. Purified rAbpA, along with purified amylase, interacted with and precipitated Gtfs from culture supernatants of both *S. gordonii *and *S. mutans*. The presence of amylase and/or rAbpA increased both the sucrase and transferase component activities of *S. mutans *Gtf-B. Enzyme-linked immunosorbent assay (ELISA) using anti-Gtf-B antibody verified the interaction of rAbpA and amylase with Gtf-B. A *S. gordonii abp*A-deficient mutant showed greater biofilm growth under static conditions than wild-type in the presence of sucrose. Interestingly, biofilm formation by every strain was inhibited in the presence of saliva.

**Conclusion:**

The results suggest that an extracellular protein network of AbpA-amylase-Gtf may influence the ecology of oral biofilms, likely during initial phases of colonization.

## Background

Saliva-bacterial interactions influence the establishment and maintenance of the microflora of dental plaque [1-3]. Amylase is the most abundant enzyme in human saliva and is a constituent of the complex glycoproteinacious acquired pellicle that immediately forms on cleaned teeth [4-7]. Dental plaque forms on the pellicle and is responsible for the most common oral diseases, dental caries and periodontitis [8,9]. Amylase specifically binds with high affinity to several oral commensal streptococcal species, including *Streptococcus gordonii*, *Streptococcus mitis*, *Streptococcus parasanguinis, Streptococcus cristatus*, and *Streptococcus salivarius*, but not to *Streptococcus sanguinis *or to cariogenic streptococci including *Streptococcus mutans *and other mutans streptococci [10-13]. Amylase-binding bacteria constitute a substantial proportion of the total cultivable flora on human teeth and only colonize the mouths of animals with salivary amylase activity [14]. This suggests that the ability to bind amylase is ecologically advantageous to these bacteria for colonization of oral surfaces.

*S. gordonii *produces two amylase binding proteins: amylase binding protein A (AbpA) [15] and amylase binding protein B (AbpB) [16]. Both proteins are expressed transiently on the cell surface before release into the extracellular *milieu *in soluble form. AbpA is a 20 kDa protein that acts as the major receptor for salivary amylase binding to the bacterial surface. Mutants deficient in AbpA adhere less well to amylase-coated hydroxyapatite and appear deficient in a dynamic model of biofilm formation *in vitro *[17]. Paradoxically, however, AbpA-deficient strains of *S. gordonii *colonize the mouths of conventional rats as well or better than do wild-type (WT) strains [18]. In fact, oral colonization by AbpA-deficient mutants appears augmented when the rats are fed dietary sucrose. Furthermore, glucosyltransferase (Gtf) activity, shown to be an important colonization factor for oral streptococci [19,20], appears greater in AbpA-deficient mutants [18].

*S. gordonii *produces a single Gtf (Gtf-G) [21], while *S. mutans *produces three Gtfs (Gtf-B, Gtf-C, and Gtf-D) [22]. Gtfs synthesize extracellular glucan from sucrose and are long known as determinants of bacterial colonization of the teeth [19,20]. Gtf enzymatic action entails two steps: the hydrolysis of sucrose into fructose and glucose by its sucrase activity, and the transfer of glucosyl residues to form alpha-linked glucans by its transferase activity. Gtfs consist of two relatively conserved structural domains, an N-terminal catalytic domain and a C-terminal glucan-binding domain [23]. Although Gtfs of *S. gordonii *and *S. mutans *have amino acid homologies within the conserved domains, the enzymes differ in their requirements for acceptor molecules and ability to produce glucans with varying proportions of α-1-3 and α-1-6 glucosidic linkages [22].

It has been reported that the interaction between amylase and streptococcal Gtf on saliva-coated hydroxyapatite surfaces results in reduction in Gtf enzymatic activity and glucan formation [24]. Thus, the interaction of these enzymes may modulate the adhesion and colonization of *S. gordonii *and other Gtf-producing oral streptococci, e.g. *S. mutans*. Indeed, proteins released from one organism (e.g. *S. gordonii*) may interact with proteins released by another (*S. mutans*) within the oral biofilm milieu to modulate their function. The goal of this study therefore was to investigate the interaction of purified salivary amylase and rAbpA, alone and in combination, with Gtf of *S. gordonii *and *S. mutans*.

## Results

### Precipitation of Gtf-G and AbpA from *S. gordonii* supernatants by salivary amylase

**Figure 1:**
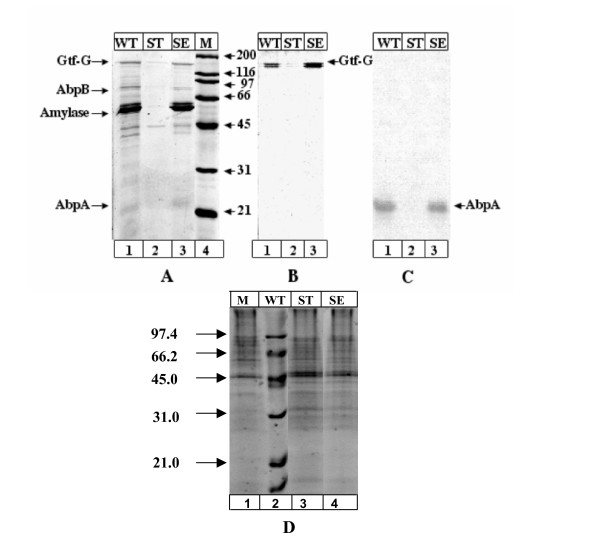
**SDS-PAGE analysis of amylase precipitates**. *Panel A*. Precipitates from 18 h culture supernatants, were boiled in sample buffer. The samples containing 3.5 μg of protein were run on 12% SDS-PAGE, followed by staining with SYPRO red in 7.5% acetic acid solution. Lane 1, supernatant of wild type cells (WT); lane 2, supernatant of AbpA-deficient strain (ST); lane 3, supernatant of AbpB-deficient strain (SE); lane 4, molecular mass standard in kDa (M). *Panel B*. Precipitates from supernatants containing 1.4 μg of protein were used for Gtf-G activity assay. Samples were run on a 12% gel and stained for GTF activity. Lane 1, supernatant from wild type culture (WT); lane 2, supernatant from AbpA-deficient cells (ST); lane 3, supernatant from AbpB-deficient cells (SE). *Panel C*. Precipitates from supernatants containing 1.4 mg of total protein were used for Western Blot using polyclonal anti-AbpA antibody. Lane1, 2, and 3, supernatants from wild type (WT), AbpA-deficient (ST), and AbpB-deficient cells (SE), respectively.

Our previous studies revealed that the addition of purified amylase to *S. gordonii *culture supernatant resulted in the precipitation of soluble amylase-binding proteins [16]. To further investigate this interaction, purified amylase was added to cell-free culture supernatants of WT and mutant strains of *S. gordonii*. While addition of amylase did not induce precipitation of proteins from the AbpA^- ^strain's culture supernatant, it did so from supernatants of both the AbpB^- ^and WT strains (Fig. 1A; lanes 1, 2 and 3), indicating that AbpA is essential for precipitation. SDS-PAGE analysis of the precipitate from WT culture supernatant showed the presence of several proteins, including AbpA and AbpB, as well as Gtf-G (Fig. 1A; lane 1). Precipitation of Gtf-G and other amylase-binding proteins by purified amylase was found to be dose-dependent; when amylase was added to 0, 1, 5, 10, 20, 40, 50 mg/ml, maximum precipitation appeared to occur at 40 mg/ml and greater. A sham experiment in which buffer containing bovine serum albumin (BSA) alone was added in place of amylase did not precipitate any proteins (data not shown).

In contrast, SDS-PAGE analysis of 10-fold concentrated and dialyzed spent culture supernatant showed no protein bands above 20 kDa on. Further, while amylase induced protein precipitation from cell free culture supernatants, it did not do so from dialyzed culture supernatant (data not shown). This observation suggested that ionic strength is also an important determinant for amylase – induced precipitation.

Gtf-G activity assessment of precipitates from supernatants following addition of amylase revealed the essential lack of Gtf-G activity from the AbpA^- ^strain (Fig. 1B; lane 2). The presence and absence of AbpA in the precipitate was verified by immuno-blotting using rabbit anti-AbpA antibody (Fig. 1C). MALDI-TOF analysis of proteins eluted from the gel identified AbpB and amylase in the precipitate obtained from WT culture supernatants. As shown in Fig. 1A and 1B, while amylase did not precipitate Gtf-G from the AbpA^- ^supernatant, it did so from supernatants of both the AbpB^- ^strain and the WT. The presence of Gtf-G activity in the precipitates obtained from the supernatant of AbpA positive strains (WT and AbpB^- ^mutant), and the absence of Gtf-G activity that could be harvested from the AbpA^- ^supernatant, indicated that amylase together with AbpA interacted with and precipitated Gtf-G. Figure 1D shows an SDS-PAGE stained with SYPRO of alcohol precipitates from culture supernatants from each strain (WT, AbpA^- ^and AbpB^-^). These precipitates show many more bands than found in the amylase precipitates from each strain, suggesting selective precipitation of only a few bands by amylase.

### Inhibition of (^125^I) α-amylase-bacterium interactions by rAbpA

Previous studies have shown that α-amylase binds non-covalently to the *S. gordonii *surface with high affinity, and that AbpA acts as the major cell surface receptor for amylase binding [10,15,17,25]. To confirm the role of AbpA as this receptor, rAbpA was expressed and purified from *E. coli *as described in the Experimental Procedures section. Far western blot analysis of purified rAbpA using salivary amylase as a ligand confirmed the retention of its amylase binding activity. We further quantitatively measured binding of labeled amylase to the WT cell surface in the presence of purified rAbpA. The dose-dependent inhibition of (^125^I)-amylase to the bacterial surface following preincubation with rAbpA confirmed the amylase binding function of AbpA on the bacterial surface (data not shown).

### Amylase precipitates Gtf-G in the presence of rAbpA

**Figure 2:**
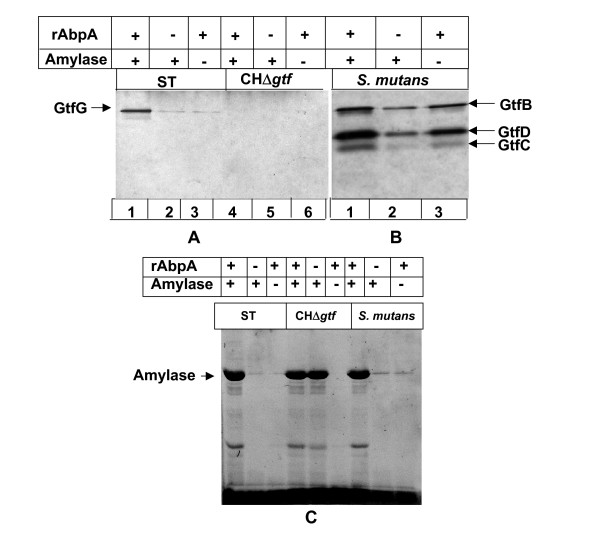
**Gtf enzyme activity assay**. Overnight culture supernatant of strains in question, containing 3 μg total protein were used to precipitate Gtfs. To it, amylase and/or rAbpA was added to a final concentration of 50 μg/ml each. After 2 h at room temperature, precipitates were collected by centrifugation and resuspended in sample buffer and Gtf activity evaluated on SDS-polyacrylamide gel. *Panel A*. Lanes: 1, 2, and 3, supernatant from *abpA*^- ^strain (ST); lanes 4,5, and 6, supernatant from *Gtf-*deficient strain. *Panel B*. Lanes 1, 2, and 3, supernatant from *S. mutans*. *Panel C*. SDS-PAGE gel of precipitates from supernatants stained with SYPRO red. Lanes 1, 2, and 3, supernatants from AbpA-deficient strains; lanes 4, 5, and 6, supernatants from gtf-negative strains of *S. gordonii*; lanes 7, 8, and 9, supernatants from *S. mutans*.

Because detectable levels of Gtf-G were not precipitated by amylase from the culture supernatant of the AbpA^- ^(strain ST) strain (Fig. 1A and 1B), and amylase-binding proteins are not produced by *S. mutans *[10,12,26], we used cell-free culture supernatants of the AbpA^- ^strain of *S. gordonii*, and from *S. mutans *10449S, for a Gtf-amylase precipitation assay in the presence or absence of exogenous purified rAbpA and/or amylase. Amylase, when added in the presence of rAbpA, precipitated Gtf-G from the AbpA^- ^strain (strain ST) supernatant (Fig 2A) and rAbpA augmented amylase precipitation of Gtf-B, Gtf-C, and Gtf-D of *S. mutans *(Fig. 2B). However, amylase or rAbpA, singly, precipitated a small amount of GtfG and Gtf-C from the supernatants of these strains. As expected, the supernatant from a Gtf^- ^mutant of *S. gordonii *did not form any precipitate under any of these conditions (Fig. 2A). Image scanning of SDS-PAGE activity gels using AlphaImager software (Alpha Innotech, San Leandro, CA, USA), showed that addition of both amylase and rAbpA to the culture supernatant from the WT resulted in a 1.5- to 3.0-fold increase in the Gtf-G and Gtf-C activities by comparison with the single addition of amylase or rAbpA. Increased activities of Gtf-B, Gtf-C and Gtf-D were also observed when both amylase and rAbpA were added (Fig. 2B). Further, SYPRO red staining of SDS-PAGE of amylase precipitates from supernatants showed that amylase in combination with rAbpA precipitated the proteins than when compared with amylase or rAbpA alone (Fig. 2C).

### Effects of amylase and rAbpA on sucrase and transferase activities of Gtf-B

**Figure 3:**
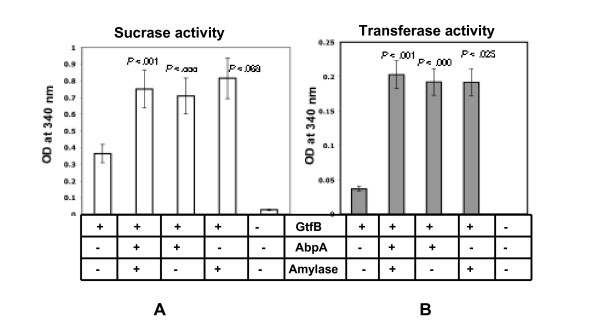
**Measurement of sucrase and transferase activities of GtfB**. Purified GtfB enzyme was incubated at 37°C overnight with sucrose in presence or absence of amylase and/or rAbpA. The amount of glucose and fructose present in the reaction mixtures were measured using the F-kit. *Panel A and B*. Sucrase activity and transferase activity of GtfB in the presence of amylase and/or rAbpA, respectively. For statistical analysis, Gtf-B activities in the presence of amylase and/or rAbpA were compared with Gtf-B alone.

Sucrase activity of the Gtf-B was increased 2- to 2.5-fold (p < 0.001), and transferase activity was elevated 4- to 6-fold (p < 0.001), respectively, in the presence of purified amylase and/or rAbpA (Fig. 3A and 3B). Under identical assay conditions, there was no effect on Gtf-B enzyme activity when BSA alone was added as a control (data not shown). In addition, rAbpA or amylase alone showed little transferase or sucrase activities (Fig. 3). Gtf-B transferase activity in the presence of rAbpA and/or amylase was significantly greater when compared with Gtf-B alone (*p *≤ 0.025). Gtf-B sucrase activity was also elevated in presence of rAbpA or rAbpA supplemented with amylase (*p *≤ 0.01). However, no additive/synergistic effects on sucrase or transferase activities were noted when Gtf-B was incubated together with both rAbpA and amylase.

### Binding of rAbpA and amylase with Gtf-B

**Figure 4:**
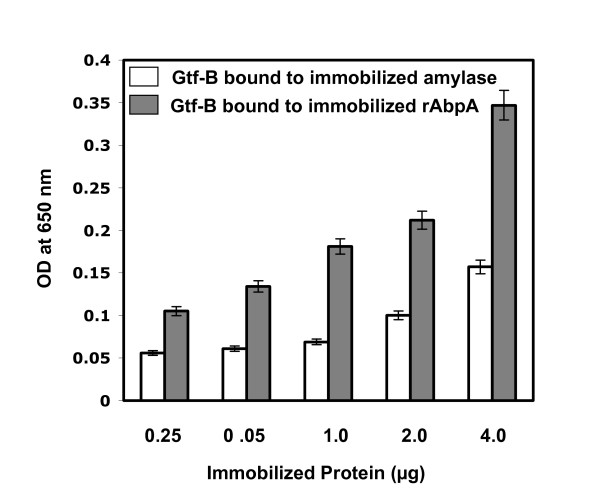
**Binding of rAbpA and amylase with GtfB**. For coating antigens onto wells of ELISA plate, 4, 2, 1, 0.5, and 0.25 μg of either amylase or rAbpA were used. To each well, 1 μg of ligand protein (Gtf-B) was added.

The above described results suggested that intermolecular interactions occur between Gtf, AbpA and amylase, and that either amylase or rAbpA alone stimulated activity of Gtf-B, suggesting that these molecules interact with each other. These interactions with Gtf-B were verified using ELISA to measure binding of soluble Gtf-B to immobilized rAbpA or amylase in a dose dependent manner (Fig. 4). Control experiments were also performed demonstrating that neither purified amylase nor rAbpA were able to bind to BSA-coated wells (data not shown). Immobilized rAbpA or amylase also interacted with Gtf-B (Fig. 4) in a manner directly proportional to the amount of immobilized proteins, supporting the interaction of rAbpA and amylase with Gtf-B.

### Biofilm production by S. gordonii *WT *and the AbpA^- ^mutant

**Figure 5:**
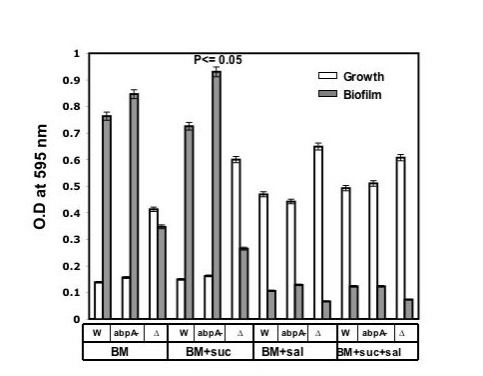
**Growth and biofilm formation of wild-type, *abp *A and Δ*gtf *mutants of *S. gordonii***. Bacteria were grown in BM or BM with 1% sucrose and/or 25% saliva. Growth and biofilm formation were measured under CO_2 _enriched condition. All assays were performed  eight times, and mean values and standard deviations are shown..

*In vitro *experiments described above indicated that rAbpA interacts with Gtf and increases Gtf enzymatic activity. They suggest a potential role for AbpA as a modulator of *S. gordonii *and *S. mutans *colonization in the oral cavity. To further explore this, we performed *in vitro *biofilm experiments to compare the wild-type, AbpA^- ^and Gtf-G negative mutants, in the presence and absence of sucrose and/or saliva (Fig. 5). While the addition of sucrose did not result in differences in final growth between the WT and AbpA^- ^mutant strains as determined by measurement of optical density, sucrose did increase biofilm formation in the AbpA^- ^mutant when compared to the WT (Fig. 5). Surprisingly, biofilm formation by every strain was inhibited in the presence saliva (Fig. 5). Furthermore, the *abp*A mutant showed a tendency to produce more biofilm growth than the WT in the absence of sucrose.

## Discussion

The results of these studies demonstrate that salivary amylase complexes with Gtf in the presence of AbpA. Addition of exogenous purified amylase to culture supernatants precipitates Gtfs from both wild type and a AbpB^- ^mutant of *S. gordonii*, but not from a AbpA^- ^mutant of *S. gordonii*, or from *S. mutans *(known not to bind amylase or produce any amylase-binding-protein). Both purified rAbpA and amylase increased the level of Gtf enzyme activity. Previous studies reported that streptococcal Gtfs interact with salivary amylase immobilized on the surface of hydroxyapatite beads, reducing Gtf activity [24]. In the present study, *S. mutans *Gtf-B in solution interacted with either soluble amylase or with rAbpA with resultant increased enzyme activity. We chose to study Gtf-B since it is strongly associated with cariogenicity of *S. mutans*, it was readily available in pure form, and could potentially interact with AbpA within the multispecies dental plaque biofilm environment. Because the presence of both AbpA and amylase did not synergistically stimulate Gtf activity, we surmise that AbpA and salivary amylase may bind to the same (or nearby) site(s) on Gtf to modulate its enzymatic activity. This is consistent with the known homology of amylase and the Gtf active site regions as previously reported [27].

The *abp*A-deficient mutant formed quantitatively greater biofilm mass than the WT when grown statically on polystyrene surfaces in both the presence and absence of sucrose, consistent with *in vivo *findings that the AbpA-deficient mutant strain is a better colonizer of rodent teeth than is the WT strain [18]. The present findings contrast with our previous report that AbpA-deficient strains of *S. gordonii *were less able to form *in vitro *biofilm than the WT strains [17]. These earlier studies, however, were performed using a flow biofilm model in the presence of 25% saliva, and in the absence of sucrose. It is also interesting to note that the inclusion of saliva in the present model inhibited biofilm formation by all of the bacterial strains tested. This is in contrast to previous studies that show that *S. gordonii *initial adhesion to hydroxyapatite is promoted by saliva [28]. The differences noted in results from the various *in vitro *models reinforces the limits of *in vitro *model systems, and the necessity for caution in their interpretation.

Our previous study found that *S. gordonii *strains deficient in AbpA colonized rats fed a sucrose-free starch diet better than their wild-types, and that this differential advantage in colonization was enhanced in rats fed a high sucrose diet [18]. We interpreted these results at that time by suggesting that AbpA may interact with Gtf to reduce its enzymatic activity, consistent with a previous report [24]. The present results demonstrate that purified AbpA and amylase can interact with Gtf to increase enzymatic activity. It is likely that present *in vitro *models of biofilm formation are at best a gross simplification of the diverse and complex events occurring *in vivo*. Thus, a possible explanation for this paradoxical result is that the interaction of amylase and AbpA with Gtf may serve to alter the chemical nature of the resulting glucan products to modulate colonization. Indeed, a previous study found that glucan synthesized by immobilized Gtf-B in the presence of starch hydrolysates was less susceptible to hydrolysis by fungal mutanase than was glucan made by immobilized Gtf-B in the absence of starch hydrolysates [24]. Furthermore, glucan production by Gtf-B associated with streptococci immobilized on saliva-coated hydroxyapatite was also enhanced in the presence of starch hydrolysates. Some bacteria displayed higher adhesion activities for the glucan made in the presence of the hydrolysates. The present findings suggest that an amylase-AbpA-Gtf complex may alter the composition of resulting glucans to affect adhesion, biofilm formation and oral bacterial colonization. This possibility deserves further study.

Of course, *in vitro *experiments performed with purified molecules may not reproduce the *in vivo *situation. It is possible that additional interactions that occur *in vivo *influence colonization. Nevertheless, the present results suggest that a protein secreted by *S. gordonii *(AbpA), along with a host protein (amylase), may together affect the function of *S. mutans *Gtf to influence perhaps *S. mutans' *colonization. This could point to a potential mechanism by which non-mutans streptococci might influence *S. mutans *ecology.

Stimulation of Gtf activity, as seen in the present report, is likely the result of the physical interaction between amylase/rAbpA, with subsequent conformational change in Gtf structure. Taken together, the present observations demonstrate intermolecular interactions between amylase, rAbpA, and Gtf, yielding modulation of Gtf activity in the complex. Studies are now proceeding to examine further the structural basis of the amylase-AbpA-Gtf interaction.

## Conclusion

These studies demonstrate that salivary amylase complexes with Gtf in the presence of AbpA. Addition of exogenous purified amylase to culture supernatants precipitates Gtfs from both wild type and a AbpB^- ^mutant of *S. gordonii*, but not from a AbpA^- ^mutant of *S. gordonii*, or from *S. mutans *(known not to bind amylase or produce any amylase-binding-protein). Both purified rAbpA and amylase increased the level of Gtf enzyme activity. The results suggest that an extracellular protein network of AbpA-amylase-Gtf may influence the ecology of oral biofilms, likely during initial phases of colonization.

## Methods

### Bacterial strains and culture conditions

*S. gordonii *strains Challis-S (wild type, a spontaneous streptomycin resistant mutant of strain Challis), and its AbpA-(strain ST) and AbpB-deficient (strain SE) mutants,[16,17], a Gtf-G-deficient mutant (CHΔ*gtf*) [29] and *S. mutans *10449S, a spontaneous streptomycin resistant mutant of strain NCTC-10449, were recovered from frozen stocks on tryptic soy blood agar and incubated overnight in a candle jar. Streptococci were routinely cultured in tryptic soy broth (Difco, Detroit, MI. USA) containing 0.5% (w/v) yeast extract (TSBY) overnight at 37C. *Escherichia coli *was grown in Luria-Bertani broth (LB) with constant shaking at 37°C and maintained on LB agar.

### Construction of the AbpA expression system

The expression vector, pETBlue-1 Blunt (Novagen, Madison, WI. USA), was used to clone and express the *abpA *of *S. gordonii*. A 1.7 kb *Xba*I DNA fragment containing *abpA *was purified from pCR2kb-7 [15] and then inserted into pETBlue-1 Blunt vector, resulting in plasmid p101-1. The 1.6 kb *EcoR*I fragment from p101-1 was purified and digested with ApoI. The resultant 758 bp *Apo*I fragment was eluted from 2% (w/v) agarose gel and cloned into the pETBlue-1 blunt vector, resulting in p102-4, the *abpA *expression clone. All recombinant plasmids were introduced by chemical transformation into NovaBlue Singles™ Competent Cells (Novagen), and selected on LB agar supplemented with ampicillin (100 μg ml^-^). The orientations of cloned genes were confirmed by restriction enzyme analysis. Plasmids containing inserts of correct size and orientation were purified using Wizard Plus SV Minipreps DNA Purification System (Promega, Madison, WI, USA), and the fidelity of the cloned region verified by sequencing.

### Expression of AbpA in E. coli

The *abpA *plasmid p102-4 was transformed into Turner™(DE3)pLacI Competent Cells (Novagen) which were grown to 1.0 OD_600 _in 250 ml LB supplemented with ampicillin (50 μg ml^-1^) and 1% glucose. The cells were induced by the addition of 1 mM isopropyl-D-thiogalactopyranoside (IPTG) for 2 h at 37°C with constant shaking. Cells were harvested by centrifugation at 4000 × g for 15 min at 4°C, and the cell pellet (~2.3 g wet weight) was stored frozen at -20°C. The induced *E. coli *cell pellet was suspended in 50 ml of EasyLyse™ Bacterial Protein extraction solution (5 mM Tris-HCl (pH 7.5), 0.5 mM EDTA, 0.5% Triton X-100, 0.1 mM MgCl_2 _and 20 μl EasyLyse™ enzyme mix) (Epicentre, Madison, WI, USA). The insoluble cellular debris was removed by centrifugation (10,000 × g) at 4°C for 10 min and the soluble supernatant transferred to a clean tube. The total supernatant protein concentrations were estimated using BCA reagent (Bio-Rad, Hercules, CA, USA), and the supernatant containing the rAbpA was stored at -20°C.

### Purification of rAbpA

rAbpA was concentrated by anion exchange Macro-prep DEAE methacrylate (Biorad) in a 5 ml column equilibrated with 10 mM Tris buffer, pH 7.3. Crude supernatant (10 ml) containing 2 mg protein ml^-1 ^of the *E. coli *extract that included rAbpA was loaded onto the column and eluted by a 0 to 1 M linear NaCl gradient. Twenty-5 ml fractions were collected and monitored at 280 nm. All fractions were further analyzed by 12% SDS-PAGE [30]and stained with either 0.1% (w/v) Coomassie brilliant blue R-250 or SYPRO red (Cambrex, Rockland, MI, USA). N-terminal amino acid sequencing was performed to verify the amino acid sequence of rAbpA (data not shown). Purified proteins were lyophilized and stored for further studies.

### Western immunoblotting or amylase ligand binding assay

Western blotting [31] was performed by electrotransfer of proteins from SDS-PAGE gels to Immobilon-P membranes (Millipore, Bedford, MA, USA). For the amylase ligand-binding assay, after blocking with 5% (w/v) non-fat milk in TBST, membranes were incubated with a solution of purified amylase (Sigma, St. Louis, MO, USA; Cat # A-1031. A stock solution of 10 mg/ml was made in deionized water and stored at -20°C.) in TBST for 30 min. After washing, the blots were incubated with either polyclonal rabbit anti-AbpA [25] or polyclonal anti-human α-amylase (Sigma), washed, and then incubated with goat anti-rabbit IgG conjugated with alkaline phosphatase (Promega). Finally, the blots were developed using the ProtoBlot Western Blot AP system (Promega).

### Matrix assisted laser desorption-ionization-time-of-flight analysis

The pure, lyophilized rAbpA was dissolved in water. Approximately 1 p-gram of the protein was mixed with sinapinic acid solution (10 mg ml^-1 ^in 60% acetonitrile, 0.1% tri-fluoroacetic acid), spotted onto the sample analysis plate, and dried. The protein was then analyzed by matrix assisted laser desorption-ionization-time-of-flight (MALDI-TOF) mass spectrometry (Bruker Daltonics, Bremen, Germany). Mass spectra were acquired with an accelerating voltage of 19 kV using linear mode. A molecular mass of 18121.024 Da was observed for rAbpA by MALI-TOF (data not shown). The instrument was externally calibrated with carbonic anhydrase and bovine serum albumin.

For mass fingerprinting, about 100 μg of the pure lyophilized protein was resuspended in 20 μl of 0.25 mg ml^-1 ^sequence grade trypsin (Promega) in water and incubated overnight at 37°C. Mass spectra were obtained using reflection mode. The monoisotopic masses obtained for the peptides were in the range of 919.523 to 1985.091. The molecular masses of the digested peptides obtained by MALDI-TOF showed coverage of 47% and were matched with the peptide masses obtained using Peptidemass software (data not shown). The instrument was calibrated externally with ACTH and angiotensin.

### Inhibition of binding of (^125^I)α-amylase by rAbpA to *S. gordonii*

These experiments were performed essentially as previously described (Scannapieco, et al. 1989). Briefly, freshly grown *S. gordonii *cells were suspended in PBS containing 0.01% (w/v) lipid-free bovine serum albumin (BSA) to a final concentration of 3 × 10^9 ^cells ml^-1^. Experiments were performed in polypropylene micro-centrifuge tubes pre-coated with 0.1% (w/v) BSA to reduce nonspecific binding of ligand with the tube wall. Reaction mixtures of 0.1 ml, containing 1.1 pmole of (^125^I) α-amylase in PBS, and various amounts of rAbpA were incubated at room temperature for 30 min. To this mixture bacteria suspended in PBS were added to final volume of 0.5 ml, mixed gently, incubated for 30 min, and the reaction terminated by centrifugation at room temperature followed by three 1 ml washes with PBS. The amount of radioactivity, reflecting amylase bound to the bacteria, was measured with a gamma counter (Model 5500; Beckman City, CA, USA).

### Precipitation of amylase-interactive proteins from *S. gordonii *culture supernatants

Supernatants were collected from overnight cultures by centrifugation at 5000 × g for 10 min at room temperature and passed through a 0.2 micron filter. Precipitation of proteins was induced by the addition of purified salivary amylase (50 μg ml^-1^) to the supernatant (Li *et al*., 2002). After incubation at room temperature for 2 h, the precipitate containing amylase and streptococcal proteins was recovered by centrifugation at 5000 × g for 10 min, resuspended in sample buffer (0.06 M Tris-HCl, pH 6.8, 10% (v/v) glycerol, 2% (w/v) SDS, 0.05% (v/v) 2-β-mercaptoethanol and 0.00125% bromophenol blue, and boiled for 3 min. Subsequently, the precipitate components were resolved on 12% SDS-PAGE, stained with SYPRO RED in 7.5% (v/v) acetic acid, and photographed under UV light.

### Glucosyltranferase activity assay

Gtf activity was evaluated by both qualitative and quantitative assays. For qualitative assays, activity was determined in polyacrylamide gels as previously described [32]. Briefly, cell free supernatants were run on SDS-PAGE followed by overnight incubation of gel in 3% sucrose. The glucan bands synthesized by the GTF were visualized by staining with periodic acid and pararosanaline.

The component sucrase and transferase activities of Gtf-B were differentially quantified as previously described [33,34]. Briefly, 0.6 μg of soluble Gtf-B (kindly provided by Dr. Ann Vacca-Smith, University of Rochester, NY, USA) in 2 μl of 0.2 M sodium phosphate buffer (pH 6.0), was incubated in the presence or absence of various amounts of soluble rAbpA (2 μl) and/or amylase (2 μl in buffer) at room temperature for 5 min. To this, 50 μl of buffer and 40 μl of 1 M sucrose in water were added to a final volume of 100 μl and the reaction mixture was incubated overnight at 37°C. The amounts of free glucose and fructose in the reaction mixture were measured by an enzymatic-redox reaction train using hexokinase, glucose-6-phosphate dehydrogenase, 6-phosphoglucose isomerase, and NADP ^+ ^(F-Kit, R-Biopharm, Mannheim, Germany). The amount of resulting fructose in the reaction mixture represents sucrase activity; the difference between the amounts of free glucose and free fructose in the reaction mixture corresponds to the amount of glucosyl residues transferred to glucan, and thus the transferase activity [35]. Means (± S.E.) were used to describe the enzyme activities.

### Interaction of rAbpA and amylase with Gtf-B of S. mutans

ELISA was used to verify the interaction of rAbpA and amylase with Gtf-B. Two-fold dilutions of Gtf-B or rAbpA were coated onto wells of ELISA plates. The plates were washed and blocked as per manufacturers instructions (Kirkegaard and Perry, Gaithersburg, MD). To each well, 1 μg of purified amylase, Gtf-B or rAbpA was added and incubated at room temperature for 2 hr. After washing, the samples were probed with polyclonal anti-Gtf-B (kindly provided by Dr. Ann Vacca-Smith), anti-AbpA (25), or anti-amylase antibodies, as appropriate. The plates were then incubated, washed, and probed with the secondary antibody provided in the ELISA kit. The plates were developed using the kit substrate according to the manufacturer's instructions. Following color development, which was proportional to the amount of target protein interacting with the protein bound to the plate, the OD of the solution was read at 630 nm in a plate reader.

### Biofilm assay

Biofilm experiments were performed in round-bottomed polystyrene microtiter plates (Nalge Nunc International, Rochester, New York, USA). Plates containing 200 μl of biofilm media (BM) [36] in the presence or absence of 1% sucrose and/or 25% filter sterilized saliva per well were inoculated with either WT or mutant strains of *S. gordonii *(5 × 10^7 ^CFU per well) from an overnight culture grown in TSBY. After 20 h of incubation at 37°C, resultant bacterial growth in each well was quantified by measuring the absorbance at 595 nm. The wells were then washed with PBS and the biofilm that formed on the walls of the wells was fixed for 5 min by adding 200 μl of methanol at room temperature. Following air drying, 100 μl of 0.1% crystal violet (CV) solution was added to each well. After 5 min, the wells were rinsed three times with 200 μl of distilled water and air-dried. To each well, 200 ml of 95% ethanol was added and placed in a rocker for 15 min. The CV eluted from the biofilm was then quantified by measuring the absorbance at 630 nm using an ELISA plate reader.

### Statistical analysis

Statistical analyses were conducted using SPSS software, version 11. Analyses of the enzyme activity were done using the two-sided t-test. Differences were considered significant when a P value of 0.05 was obtained.

## Abbreviations

AbpA, amylase-binding-protein A; AbpB, amylase-binding-protein B; rAbpA, recombinant amylase-binding-protein A; BM; biofilm medium; BSA, bovine serum albumin; CV crystal violet; ELISA, enzyme-linked immunosorbent assay; Gtf, glucosyltransferase; kDa, kilo Dalton; MALDI-TOF, matrix-assisted laser desorption-ionization-time-of-flight; TBST, tris buffered saline with Tween 20; WT, wild-type.

## Authors' contributions

BC contributed to the design of all experiments, performed cloning, recombinant protein expression, characterization of recombinant protein, precipitation and analyses of protein complex, Western blotting, sequence analysis, MALDI-TOF, glucosyl transferase activity assay, biofilm experiments, participated in the analysis and interpretation of data, co-drafted and co-wrote the manuscript. JR purified recombinant protein. MMV assisted with writing the manuscript and supplied the gtf-negative strain. JMT participated in the interpretation of data, and contributed to writing the manuscript. FAS conceived of the project, advised in the design of experiments, co-drafted and co-wrote the manuscript. All authors read and approved the final manuscript.
